# The Girdlestone situation: a historical essay

**DOI:** 10.7150/jbji.36618

**Published:** 2019-09-18

**Authors:** C.M. Vincenten, T. Gosens, J.C. van Susante, M.P. Somford

**Affiliations:** 1 Department of Orthopaedic Surgery, Elisabeth-Tweesteden Hospital, Tilburg, The Netherlands; 2 Department of Orthopaedic Surgery, Rijnstate Hospital, Arnhem, The Netherlands

**Keywords:** Hip resection arthroplasty, Girdlestone

## Abstract

The eponymous term 'Girdlestone situation' originally referred to an excision of the femoral head in case of an acute pyogenic infection of the hip, described by Gathorne Robert Girdlestone in 1945. Over time the procedure and the indication to perform it have significantly changed. This article presents a short biography of Girdlestone with a concomitant report on investigating the evolution of the indication and technique of the Girdlestone situation from the first description up to contemporary literature.

## Introduction

Before the introduction of antibiotics and arthroplasty the treatment options for infectious or post-traumatic lesions of the hip were limited. One common option, when surgery was possible, was to resect the femoral head to relieve the painful or infected joint. An early example of head and neck resection of the femur was performed in 1818 by Anthony White on a 9-year-old child with septic non-union of a fracture of the hip.

In 1928 Gathorne Robert Girdlestone, an English orthopaedic surgeon, described excision arthroplasty of the hip as a salvage procedure for septic arthritis 1. The resulting clinical situation was later eponymously named the 'Girdlestone situation'.

Eponymous terms are easy shorthand but are subject to interpretation, and the supposed meaning of an eponymous term might differ between professionals 2. It is therefore important to establish the origin of eponymous terms and correlate this to contemporary use. Although the eponymous term 'Girdlestone procedure/resection arthroplasty' is well recognised by orthopaedic surgeons 3, the origin of this surgery and the way its indication and technique have changed over time is not well documented. The technique for head and neck resection has changed, as has the indication to perform this surgery. It seems useful to review these changes and propose a proper description of the Girdlestone resection arthroplasty (GRA) as is practised these days.

## Material and Methods

By using the original publication we retrieved the original description of the femoral head resection. We also conducted biographical research on Girdlestone. Finally, a literature review was conducted into the eponymous procedure.

A literature search in PubMed was conducted on 30 October 2018 on the Girdlestone procedure. The electronic databases Embase, Pubmed, Cochrane Register of Clinical Trials, and Google scholar were used to identify relevant studies up to 30 October 2018. The following search strategy was used in Embase: ((girdlestone* OR ((Hip OR 'femur head' OR 'femoral head' OR cox) NEAR/6 (arthroplast* OR hemiarthroplast* OR hemiarthroplast* OR prosthe* OR replacement*) NEAR/6 (excision OR resection*)) OR ((thr OR tha) NEAR/6 (excision OR resection*))):ab,ti) AND [english]/lim NOT ([animals]/lim NOT [humans]/lim). A similar search strategy was applied in the other databases listed above. References in reviews and full-text articles were screened to retrieve more studies that could be eligible for this literature study.

We analysed the articles and in chronological fashion are presenting the changes that occurred over time. We added 'historical' papers that were missing from our search strategy. The article results are presented in a table and flow diagram according to the Prisma guidelines (Figure 4). We correlate the changes of the GRA with the concurrent progress in surgery at the time.

## Results

Our PubMed/Medline search identified 1441 articles. After excluding articles that were not relevant to Girdlestone resection arthroplasty and any duplicates, 15 articles remained. By checking the references manually and finding historical papers, an additional 12 articles were identified (Figure 4).

### Life of Girdlestone

**Figure 1:**
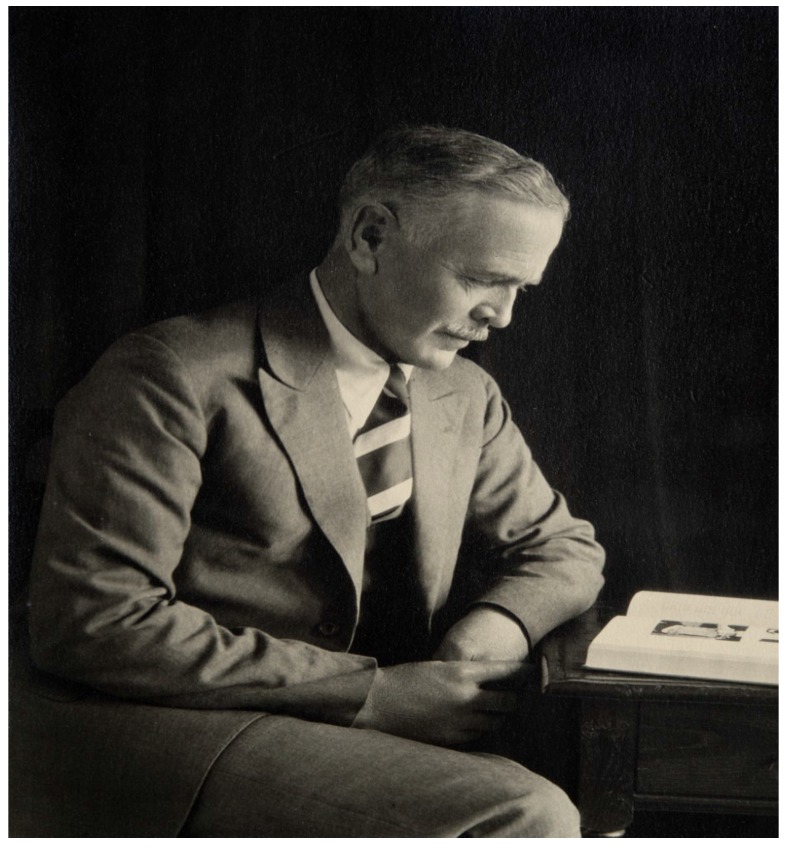
** Gathorne Robert Girdlestone (1881-1950).** (Reproduced with permission of the Nuffield Department of Orthopaedics, Rheumatology and Musculoskeletal Sciences)

Gathorne Robert Girdlestone (8 October 1881 - 30 December 1950) was born in Oxford. After attending the Charterhouse School from 1896, he started clinical training in 1905 at St Thomas Hospital in London. In 1909 he married Ina Mabel Chatterton - the marriage would remain childless. When he started practicing surgery he met Sir Robert Jones, who would become a close friend and stimulated an interest in orthopaedic surgery. Gathorne became a Fellow of the Royal College of Surgeons on 1 December 1911 (Figure 1).

Girdlestone, or GRG as he was referred to by his friends, returned to Oxford during the First World War, where he was appointed Captain of the 3rd Southern General Hospital. After expanding the capacity for casualties the facility where he worked became known as the Oxford Orthopaedic Centre in 1916. After the war Girdlestone remained in charge of the hospital. A plaque commemorating the former military hospital states that in 1922 fewer war pensioners and crippled children were being admitted. That year it became the Wingfield Orthopaedic Hospital.

With his appointment as Nuffield Professor of Orthopaedic Surgery, in 1937 Girdlestone became the first professor of this specialty in Britain. In 1939 he retired from his chair but continued to be involved with the hospital; this is apparent from his help to launch the scheme for the Nuffield Orthopaedic Centre in 1949. Gathorne and Girdlestone roads, in the vicinity of the Nuffield Orthopaedic Centre, freshly preserve his memory up to his day. On 30 December 1950 GRG died at St Bartholomew' Hospital in London at the age of 69 4, 5.

### The technique (Girdlestone resection arthroplasty)

**Figure 2:**
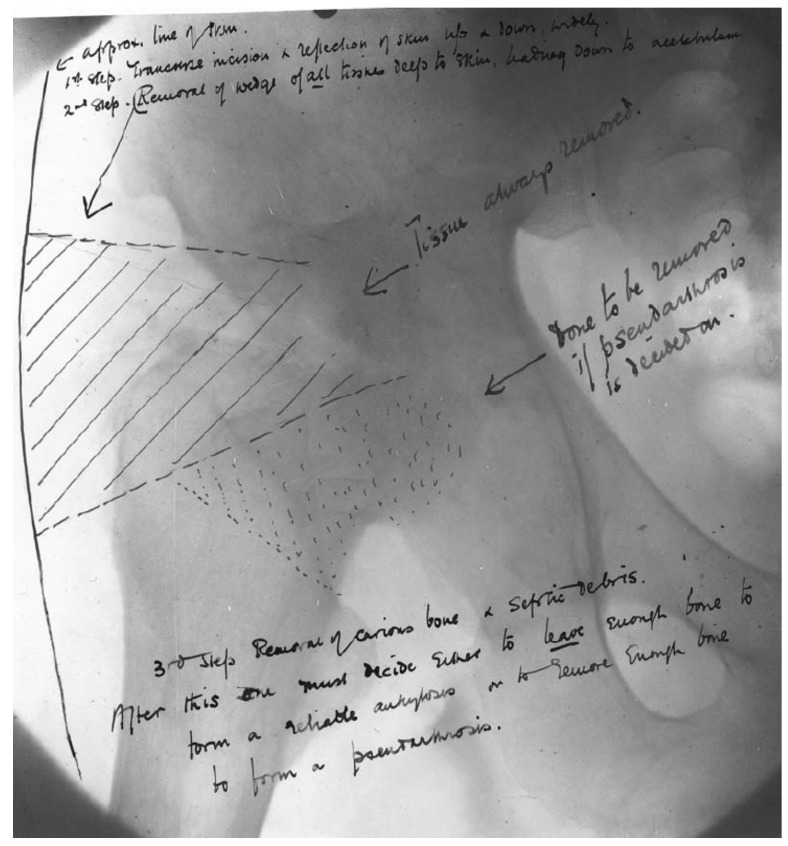
** Original drawing of the resection arthroplasty according to Girdlestone.** (Reproduced with permission of the British Editorial Society of Bone and Joint Surgery)

The earliest description of the procedure that came to be known as the Girdlestone resection arthroplasty was published in 1928 by Oxford University Press 1. The procedure is described as a radical excision to drain tuberculous hips, only performed in cases of prolonged septic infection of the hip (Figure 2). A transverse incision about 5-6 Inches (12-15cm) long with its centre near the greater trochanter is made, exposing the deep fascia and gluteal muscles. All deep tissues, including gluteal muscles and greater trochanter, were removed. A transfer wedge is removed to give free access to the diseased joint and surrounding bone. All carious bone and septic debris are removed. The cavity is packed with gauze wicks and rubber drains to ensure drainage and control secondary granulation.

The skin flaps are subsequently drawn back and stitched into the periosteum to prevent sinus-track forming, in this way reducing the pain of dressings and covering the rawer areas with excessive granulation. The subcutaneous and muscle tissues are largely covered, decreasing the granulating surface. The goal was to remove diseased and devitalised tissues, flatten down dead spaces, and leave drainage so complete and lasting as will allow the wound to heal from the bottom. A spica splintage was fitted for the patient. This is broadly explained in a letter between Girdlestone and sir Robert Jones in 1926, 2 years before the original article was published 6. What remains after this procedure is termed the Girdlestone situation.

### Use in contemporary literature

#### In relation to arthroplasty

**Figure 3:**
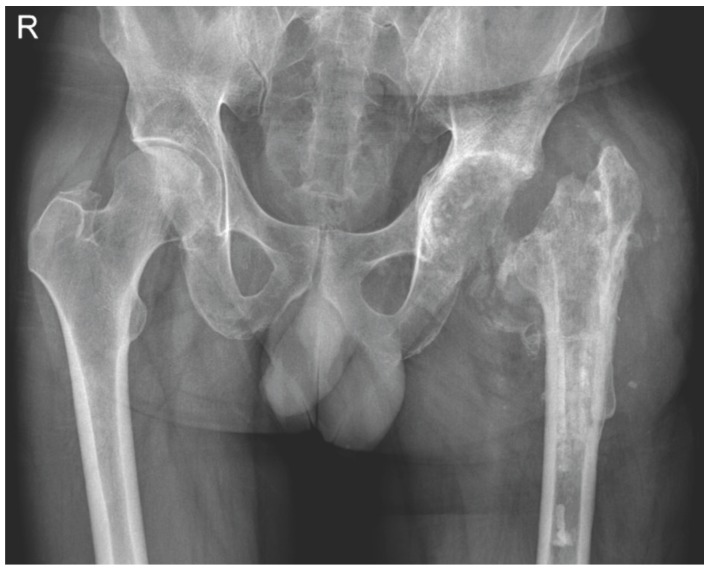
Girdlestone resection arthroplasty after an infected total hip prosthesis on the left side.

**Figure 4:**
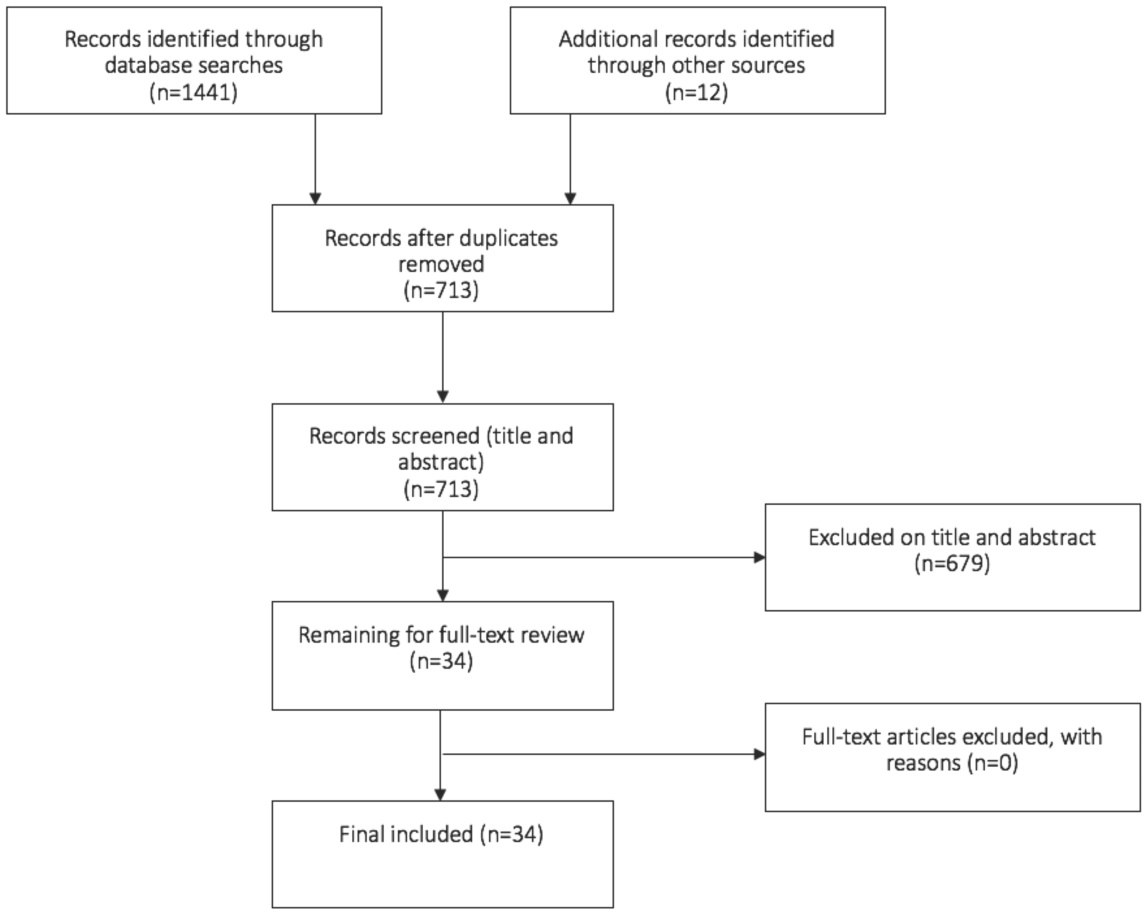
Flowchart of the included studies

The original Girdlestone resection arthroplasty (GRA), as described above, is probably no longer performed. Nowadays the eponymous term is linked to PJI treatment with a radical procedure where the hip prosthesis is completely removed, leaving only a crude articulation between femur and acetabulum (Figure 3). The proximal femur migrates 5-10 cm cranially, and finds its support just at the abductor muscles. It also exorotates due to the shape of the local pelvis 7.

In most cases, GRA is part of two-stage revision arthroplasty in the treatment of PJI. However, single-stage revision has become more popular in recent years to treat PJI 8. The Girdlestone situation could result in a permanent clinical situation when 1) bone quality or soft tissue coverage is not strong enough to insert a new prosthesis, 2) the infection cannot be controlled, or 3) patients are unfit for surgery due to e.g. multiple comorbidities 9. Functional outcome and quality of life after GRA is often impaired due to limb shortening, pain, hip instability and an inevitable need for a walking aid 10, 11.

Currently the average age for performing Girdlestone resection arthroplasty is 72 years 10. This relatively high age correlates with the average age of primary total hip arthroplasty, which is now 70-72 12.

#### In the context of cerebral palsy

In non-ambulatory cerebral palsy patients with persistent pain, chronic hip dislocation and subluxation, proximal femoral resection arthroplasty is performed as a salvage procedure for pain relief, improvement of sitting balance and perineal care 13. The procedure is frequently named after Girdlestone 14, 15, and includes extraperiosteal resection of the proximal femur from 3-4 cm below the lesser trochanter. Interposition technique involves suturing the iliopsoas and gluteal muscles to the hip capsule and covering the femoral stump by suturing the vastus lateralis to the muscles and soft tissues on the medial side 16.

## Discussion

Procedures eponymously named GRA started with the second article published by Girdlestone in 1943 in the Lancet. The article describes a related and perhaps even more radical operation for pyogenic infections 17. The cases were divided into two groups: group A included not-yet ankylosed septic infections of the hip, where the joint cavity mostly contains pus. Group B consisted of cases where ankylosis had already developed and pus had escaped from the joint, burrowing itself in the intermuscular planes. This more radical procedure was used for group B cases. The greater trochanter and all involved muscles (pectineus, adductor longus and brevis, and gluteus minimus, medius and maximus) were excised and the skin edges were sutured deep into the wound to achieve effective drainage. If necessary, the acetabulum edges were flattened.

In 1928-1950, the Girdlestone resection arthroplasty was originally indicated for pyogenic infection of the hip. These infections could be caused by fractures or gunshot wounds, or were of haematogenous origin 1, 18. After Girdlestone's death there was a growing awareness of his procedure. Excision of the femoral head was named after him and was at that time still being used to treat septic infection of the hip. The procedure was positively assessed by different surgeons 19-21. During this period, total hip arthroplasty was evolving. Smith-Petersen and Wiles, amongst others, pioneered the use of the Vitallium (a cobalt-chromium alloy) total hip replacement 22. From this point on, each year witnessed innovations in total hip replacement 23.

Total hip arthroplasty (THA) gained success in the early 1960s when it was developed by the orthopaedic surgeon Sir John Charnley. He referred to his implant as 'low-friction arthroplasty'. Charnley is considered the father of the modern THA, which uses a straight lateral approach 24, 25. Since then, the number of THAs has increased worldwide. One of the main concerns in THA is an infection as a complication 7, 9. Complete removal of the hip prosthesis without replacement is the last resort for infection treatment. In this technique, an anterolateral incision is made, followed by an incision through the fascia lata and gluteal muscles. The joint capsule is released and the femoral stem is removed. The bone marrow is cleaned and flushed. The wound is closed, leaving a drainage system. Although this does not resemble the original procedure, removal of hip prosthesis is commonly referred to as the Girdlestone resection arthroplasty 26-28.

Patterson, (1973) Haw (1976) and Bittar (1982) were the first to link prosthetic joint infection to the Girdlestone resection arthroplasty on paper 26-28. In these patients removal of the components was necessary because of infection or for mechanical reasons. All components were removed and not replaced, but the gluteal muscles were spared (group A). Around the same time several authors described the same procedure for prosthetic joint infections 29, 30.

Reported results are conflicting. In 1980 Petty had poor results for infection control and pain relief 30. However, most authors have endorsed this procedure for the management of prosthetic joint infection of the hip 29, 31, 32. Since 1984, when Bourne et al. wrote an article stating that the Girdlestone resection arthroplasty is a valuable option in the management of infected total hip prostheses, this remains the current opinion 33.

Nowadays various approaches are used, under spinal or general anaesthesia 34. The posterolateral approach is the most commonly used because of the nearly circumferential exposure of the acetabulum, the capability to displace the femoral component anteriorly and the feasibility to extend the approach towards the femur. A lower tuberculosis prevalence and the rise of antibiotics use were assumed to have the greatest influence on the discussed changes in technique and approach.

This paper provides an overview of the historical changes of the original Girdlestone resection arthroplasty. The first procedure was described in 1928 by GRG. Almost a century has gone by, and the procedure has changed. The eponym is nonetheless still being used. Currently the broad definition of a Girdlestone resection arthroplasty is removal of the head and neck of the femur without replacing anything that fills the gap, leaving a crude articulation between the proximal femur and the acetabulum.

The eponym “Girdlestone” nowadays attributed different procedures when we refer to him. This may lead to confusion in daily practice. Historically speaking, the original definition of the Girdlestone resection arthroplasty has been exposed to changes over time. This makes the eponymous term multi-interpretable and questions its correct use - which is a disadvantage when using the eponym. And yet, in contrast to most medical terms, this eponymous term is known all over the world and needs no translation.

Our study shows that since Girdlestone's first description of removal of the femoral head, the eponym is being used to describe different situations: for removal of a hip prosthesis and in femoral head ostectomy in cerebral palsy patients. Use of the Girdlestone resection arthroplasty fits both procedures well, yet we should be aware of the broad definition the eponym enjoys nowadays.

We advocate in favour of keeping the eponymous term Girdlestone situation, with the addition that it refers to a situation where there remains no formal articulation because the femoral head is resected. Whether or not the resection is primary (e.g. in case of cerebral palsy) or secondary to removal of an implant (e.g. because of infection) has no influence on the resulting situation.

## Figures and Tables

**Table 1:** Summary of articles used in this paper.

Author, year	Type of article, follow up, number of patients	Summary	Add to original search?
Girdlestone 1928^1^	Letter	About his procedure	
Oxford University Press 2006^4^	Biograph	Biograph of GR Girdlestone	Yes
Girdlestone 1943^17^	Original article	About his procedure	
Smith-Petersen et al. 1948^22^	Review	Evolution of mould arthroplasty of the hip joint	
Taylor et al. 1950^32^	Cohort, 36 months, n=93	Good outcome after GRA for advanced degenerative arthritis of the hip joint	yes
H.J.S. 1951^5^	Letter	Biograph of GR Girdlestone	yes
Nissen et al. 1952^23^	Review	Arthroplasty method by Robert and Judet	
Shepherd et al 1960^20^	Cohort 60 months, n=314	Outcome after cup arthroplasty and excision arthroplasty	
Charnley et al. 1961^24^	Clinical trial	New hip prosthesis developed by Charnley	
Scott et al. 1963^31^	Clinical trial	Outcome GRA + resection of the acetabular rim.	yes
Murray et al. 1964^21^	Cohort, 36 months, n=37	Clinical outcome after GRA	yes
Nelson et al. 1971^19^	Cohort, 40 months, n=12	Clinical outcome after GRA	
Patterson et al. 1973^27^	Cohort,18 months, n=401	Complications in total hip arthroplasty	
Glegg et al. 1974^29^	Cohort, 72 months, n=29	Outcome after GRA	
Haw et al, 1976^26^	Cohort, 120 months, n=32	Clinical outcome after hip excision arthroplasty	
Petty et al. 1980^30^	Cohort, 39 months, n=21	Outcome after GRA	
Bittar et al. 1982^28^	Cohort, n=14	Outcome after GRA	
Bourne et al. 1984^33^	Cohort, 74 months, n=33	Outcome GRA as treatment for PJI	
Baxter et al. 1986^16^	Cohort 36 months, n=4	Proximal femoral resection-interposition arthroplasty in cerebral palsy patients with hip dislocations	
Cornel et al. 1995^13^	Review	Cerebral palsy and the hip	
Garvin et al. 1995^7^	Review	Development in treatment of infection after THA	yes
Horan et al. 2005^6^	Review	Biograph of G.R. Girdlestone	
Sharma et al. 2005^11^	Cohort, 45 months, n=43	Outcome after GRA	
Chidambaram et al. 2009^12^	Retrospective cohort, n=4703	Age distribution of patients undergoing THA and TKA	yes
Root et al. 2009^15^	Review	Surgical management of the hip in the individual with cerebral palsy	yes
Basu et al. 2011^9^	Cohort, 22 months, n=24	Outcome after GRA	
Chechik et al. 2013^34^	Review (survey among 293 surgeons)	Current trends in hip arthroplasty worldwide	
Patel et al. 2015^14^	Cohort, 54 months, n=20	Femoral head excision in cerebral palsy patients	yes
Petis et al 2015^25^	Review	Overview of the current surgical approaches and its clinical outcome in hip arthroplasty	
Ramasamy et al. 2016^18^	Review	Development of surgery during world war	
Somford et al. 2017^2^	Review	Eponymous terms used in orthopaedic surgery	yes
Somford et al. 2017^3^	Review (survey among surgeons)	Eponymous terms used in orthopaedic surgery	yes
Thakrar et al. 2019^8^	Systematic review	Indications for a single stage exchange arthroplasty for chronic PJI	yes
Vincenten et al. 2019^10^	Cohort, n=63	Outcome in quality of life and health status after GRA	
